# The Effects of Continuous vs. Intermittent Prism Adaptation Protocols for Treating Visuospatial Neglect: A Randomized Controlled Trial

**DOI:** 10.3389/fneur.2021.742727

**Published:** 2021-11-19

**Authors:** Jannik Florian Scheffels, Sona Korabova, Paul Eling, Andreas Kastrup, Helmut Hildebrandt

**Affiliations:** ^1^Department of Neurology, Klinikum Bremen-Ost, Bremen, Germany; ^2^Department of Psychology, University of Oldenburg, Oldenburg, Germany; ^3^Donders Institute for Brain, Cognition and Behavior, Radboud University, Nijmegen, Netherlands

**Keywords:** neglect, rehabilitation, prism adaptation, recalibration, realignment, intermittent training, continuous training

## Abstract

Visuospatial neglect may interfere with activities of daily living (ADL). Prism adaptation (PA) is one treatment option and may involve two components: recalibration (more strategic) and realignment (more implicit). We examined whether recalibration or realignment is the driving force in neglect rehabilitation using PA. In a randomized controlled trial with two recruitment series and a cross-over design, 24 neglect patients were allocated to a continuous (PA-c) or intermittent (PA-i) PA procedure. During the PA-c condition, goggles were worn without doffing. In the PA-i condition, patients donned goggles twice (first series of patients) or three times (second series) during training to induce more recalibrations. Primary outcome parameters were performance (omissions) on the Apples Cancellation Test and ADL scores. To assess the efficacy of the PA treatment, we compared effect sizes of the current study with those from three groups from previous studies at the same rehabilitation unit: (1) a passive treatment with a similar intensity, (2) a placebo treatment with a similar intensity, and (3) a PA treatment with fewer therapy sessions. Treatment conditions did not significantly predict scores on primary and most secondary outcome parameters. However, the spontaneous ipsilesional body orientation improved only in patients receiving the PA-i condition and this improvement also appeared in patients showing a strong after-effect (irrespective of condition). Effect sizes for the Apples Cancellation Test and the Functional Independence Measure were larger for both PA treatment protocols than the historical control groups. We conclude that more recalibrations during an intermittent PA treatment may have a beneficial effect on spontaneous body orientation but not on other aspects of neglect or on ADL performance.

**Clinical Trial Registration:** German Clinical Trials Register, identifier: DRKS00018813, DRKS00021539.

## Introduction

Visuospatial neglect is a common disorder following right-hemispheric stroke and is characterized by an inability to orient to stimuli on the left side of space (1–3). It is related to difficulties in activities of daily living (ADL) and to longer treatment in a rehabilitation unit (4). Hence, neglect needs to be diagnosed carefully and treated accordingly (5). One option for treating visual neglect is prism adaptation training [PA; (6)]. During PA, patients wear wedge prisms that shift the external world about 10° rightward while repeatedly making pointing movements to visual targets (7). The prismatic shift results in a terminal error, but patients learn to compensate for it (8). After the training, patients show the characteristic after-effect: their reaching movements are biased to the left, indicating that adaptive learning has occurred.

It has been argued that the effects of PA might result from two components in the adaptation process (9–12). *Recalibration* is a strategic compensatory response needed to adjust motor commands when reaching for objects (8, 13, 14). It is seen as an *immediate reaction* to the prism-induced deviation (8, 15, 16). The second component is spatial *realignment*, which is an *automatic but slowly developing* sensorimotor learning process (13). It involves an alignment of visual and proprioceptive-motor reference frames (17) when their spatial relationships have been modified, as is the case in PA (8, 18, 19).

There is consensus that recalibration and realignment are functionally and neuronally dissociable components of the adaptation process (20–23), with recalibration being ascribed to early pointing movements after putting on the prism goggles and realignment to later trials of PA (8, 24). A recent review of neuro-imaging and neuro-stimulation studies by Panico et al. (8) proposes that realignment and recalibration processes are both mediated by distinct cerebellar and parietal areas, whereas the superior temporal gyrus and sulcus are particularly involved in realignment, and the primary motor cortex may be activated during recalibration [see also (25)]. There are also several studies specifically investigating the two processes by an active manipulation of the PA procedure (10, 12, 21–23, 26). Here, classical single-step (constant full visual shift) and multi-step (a progressive increase from no shift to full visual shift) PA conditions were compared, and the limb starting position visibility and visual feedback availability were varied. Results from these studies on healthy participants indicate that individuals who do not show recalibration still show signs of realignment and the corresponding after-effects. Performance errors and recalibration are therefore considered not to be a necessary precondition for realignment to occur (14). However, studies showing similar results in neglect patients are lacking. Hence, it remains poorly understood to what extent each of the two components drives the improvement in spatial orientation in neglect patients after PA.

Neglect as a syndrome can be viewed as an attentional-representational disorder with visuomotor components (5) and some authors assume that PA presumably works *via* circuits in the dorsal visual stream controlling attention as well as visuomotor behaviors and less *via* brain regions in the ventral stream processing perceptual components of the disorder (27, 28). However, others assume that PA treatment can specifically impact visuomotor behaviors independent from perceptual-attentional deficits (29). Regarding the motor domain, neglect can be considered as a disorder of visual-motor *calibration* (14) and it interferes with strategic control mechanisms during PA [such as pointing left of the perceived target location; (20)]. Hence, by adapting PA treatment to “overcome” this functional deficit, it may be hypothesized that offering more opportunities to learn from recalibration (i.e., using strategic processes of error correction more frequently) during treatment may produce stronger effects than commonly seen. Alternatively, based on research with healthy individuals mentioned earlier (10, 12, 21–23, 26), it may be hypothesized that a longer adaptation period without interruptions may have a beneficial effect on the realignment component and, consequently, on the outcome scores because sensory-motor learning can be fully deployed [compare (8)]. Further support for this notion comes from studies showing that longer phases of adaptation, usually achieved by executing more pointing movements, will strengthen motor learning (i.e., pointing deviations will reach an asymptote) and realignment of the visuomotor coordinates will be more intensive, indicated by larger after-effects (30, 31).

The question of whether recalibration or realignment is the major driving force for neglect symptom reduction is of considerable therapeutical relevance because the answer may have an impact on the composition of future PA training programs. These could either focus more strongly on explicit processes (i.e., strategic recalibration) by using protocols allowing for frequent donning and doffing of prism goggles or on implicit processes (i.e., realignment) with conventional continuous donning of goggles. Therefore, we carried out a randomized controlled trial (RCT) with two recruitment series. We compared two PA-conditions, one in which the goggles were worn continuously during a session (PA-c) and one in which patients donned goggles twice (in a first recruitment series) or three times (in a second series) during the training (PA-i). If recalibration is more important for the neglect outcome after PA, patients in the PA-i condition should show significantly stronger improvements than those completing the PA-c training due to additional opportunities for using explicit strategies. Otherwise, if the realignment process drives improvements more strongly, patients in the PA-c condition should show greater neglect symptom reduction.

The present study was designed to compare two PA treatment protocols and not to show whether PA is generally effective in treating neglect. Since this is also of therapeutic relevance and since several PA studies have been conducted in the same rehabilitation unit (Neurological Department of the Hospital Bremen-East), we were able to compare effect sizes from the current study with those from three earlier PA studies: 1) a passive treatment with the same training intensity (32), 2) a placebo treatment with the same intensity (33), and 3) a PA treatment with fewer therapy sessions (34). Inclusion and exclusion criteria as well as the choice and time points of neuropsychological and ADL assessments of the control groups were similar to our study (25 days between pre- and post-treatment assessments).

## Materials and Methods

### Participants

**Figure 1:**
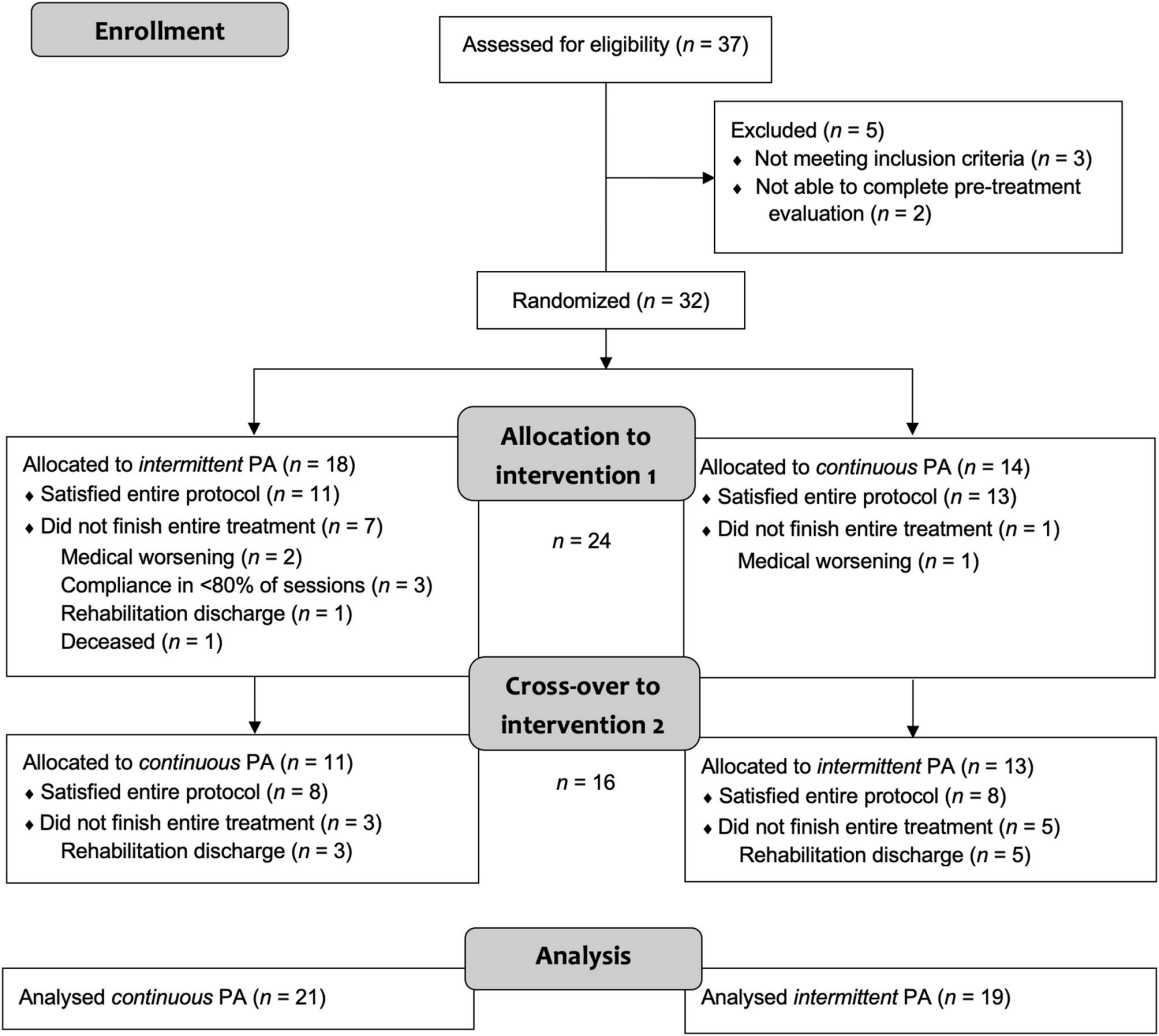
Flowchart of the sample over the course of the study (both recruitment series).

In total, 24 patients diagnosed with visuospatial neglect after right-hemispheric lesions were recruited in two series (RCT) from 2019 to 2020 at the early rehabilitation unit of the Clinic Bremen-East (Barthel Index < 30 points at admission). They all suffered from cerebrovascular disease (stroke, intracerebral bleeding, non-traumatic subarachnoid bleeding) and were randomly allotted to one of the intervention groups (first PA-i and then PA-c or the reversed order, Figure 1). Clinical characteristics are summarized in Table 1. Patients who received PA-i first were recruited on average 21.09 (*SD* = 15.53) days after disease onset; patients starting with the PA-c condition began 21.62 days (*SD* = 19.55) after onset. Inclusion criteria were the presence of left visual neglect as indicated by neuropsychological neglect tests (see below). More specifically, only patients were included who showed an impaired performance on the primary neuropsychological outcome parameter Apples Cancellation Test (35). Impaired performance was defined as more than two omissions more on the left side of the page than on the right side [page-based asymmetry score; (35)]. Moreover, they had to be able to sit in a wheelchair for at least 45 min and to participate in at least 10 of 15 planned therapy sessions. Exclusion criteria were the presence of secondary normal pressure hydrocephalus (indicated by medical report and computer tomography scans) or premorbid dementia (verified by a medical report, computer tomography/MRI scans, and reports of relatives). All patients were informed about the procedure and objective of the study and gave written consent prior to participation. The study was approved by the Bremen Medical Board's Ethics Committee and registered at the German Clinical Trials Register (IDs: DRKS00018813, DRKS00021539).

**Table 1:** Clinical characteristics of the two combined PA groups (both series and after completing both conditions of cross-over design) following an intermittent or continuous protocol.

	**PA-i (*n* = 19)**	**PA-c (*n* = 21)**
**Clinical characteristics**		
Sex (F:M)	9:10	10:11
Age (years)	61.1 ± 13.4	62.2 ± 11.7
Visual field deficit	6	8
**Etiology**		
Ischemia	7	8
Intracerebal hemorrhage	9	10
Subarachnoid hemorrhage	5	4
Mixed	3	3
**Motor impairment**		
Hemiplegia	8	7
Hemiparesis	11	14

*Values as means ± standard deviation for continuous variables*.

*PA, prism adaptation; PA-i, intermittent prism adaptation; PA-c, continuous prism adaptation*.

### Design

**Figure 2:**
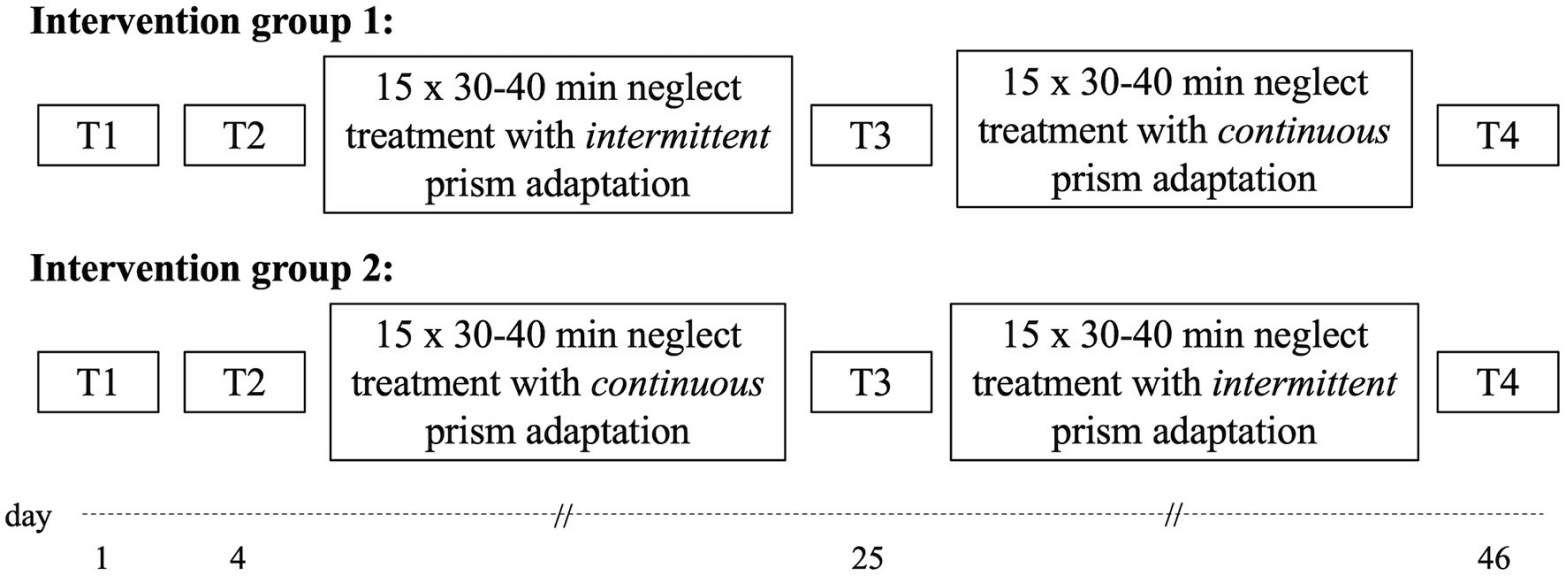
Overview of the study design.

Patients were assigned to a protocol starting either with a *continuous* (wearing the prisms continuously during the entire session), or an *intermittent* (putting them on two (first series) or three times (second series) within the same session, with doffing of prisms in-between) condition (intervention 1, see Figure 2). Therapy sessions lasting 30–40 min were offered for 15 days, with one session per day and with weekends off.

To ensure proper concealment, lists with random orders (begin with either PA-i or PA-c, followed by intervention 2) were created prior to enrolment by using a random number generation algorithm (SPSS). They were created by HH and a person external to the project chose one of them covertly. Conditions were allocated accordingly to a patient enrolled by JS or SK.

Patients were evaluated twice before the start of the treatment (T1 and T2, 3 days in-between) and 1 day after the last session of the first intervention (T3). Patients started the second intervention 1 day after T3. The next assessment took place 1 day after the last session of intervention 2 (T4). For comparing the two protocols of the first intervention, we used the results from T2 as the PRE assessment scores to control for spontaneous remission effects and from T3 as the POST treatment measurement. For the second intervention, T3 scores were used as PRE and T4 scores as POST assessments. Before conducting the study, we calculated that, with a significance level of α = 0.05 and a power of 1-β = 80%, a sample size of at least 32 patients (entire sample, two-sided tests) was necessary to ensure that at least one patient would improve performance more than 1 *SD* at a minimum.

**Figure 3:**
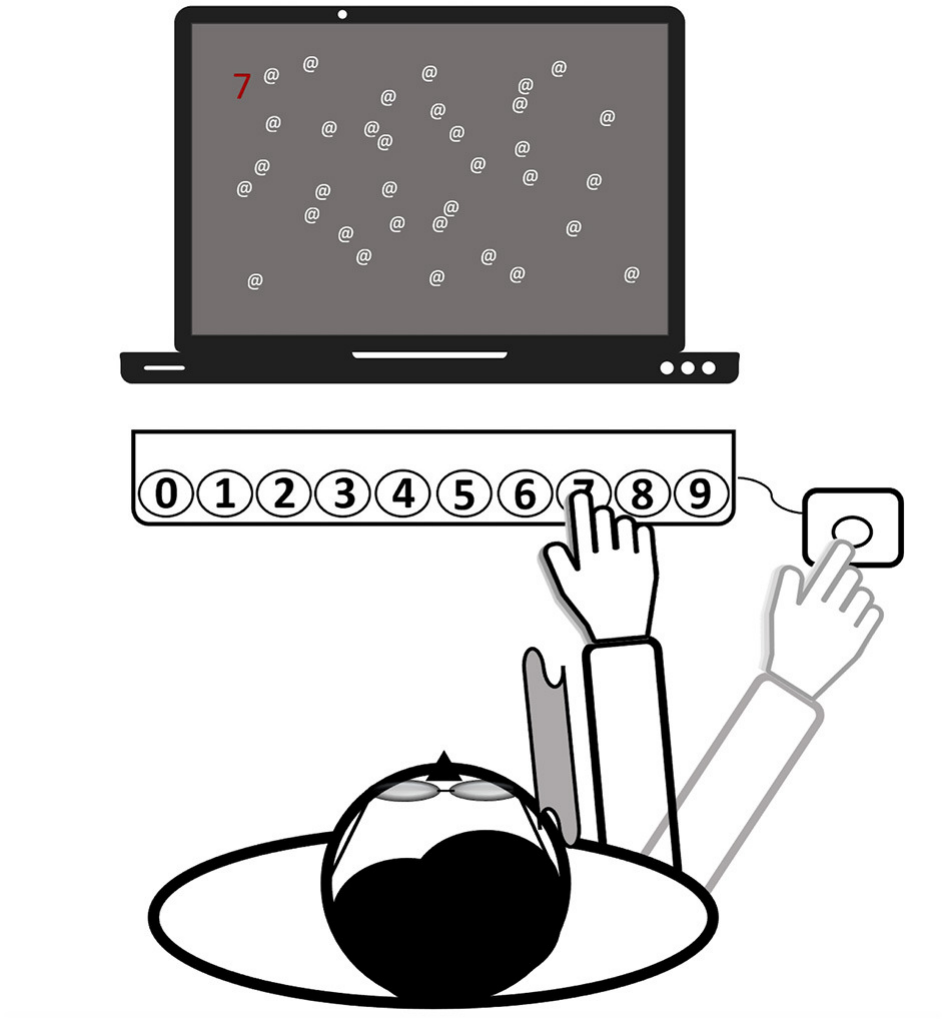
Setup of the prism adaptation treatment (all computerized tasks). Patients wear prism goggles with a cloth attached to their frame (see gray curved area), serving as a visual barrier. The depictured task is to find a randomized number among a few distractors and to press the respective button on the keyboard by making a reaching movement. Finally, for confirming the input, another movement toward an external button (right-hand side) needed to be executed. Then, the hand was laid down at this final position. The task starts again.

The setup of the PA therapy is shown in Figure 3. Patients wore wedge-shaped prism goggles that shifted the visual field 10° to the right. The patient's right visual field was restricted with a cloth (30 × 30 cm) attached laterally to the prism glasses to prevent seeing the right arm prior to movement execution (terminal exposure). In the PA-c training and in both recruitment series, patients wore the goggles continuously for 20–30 min. For the remaining 10 min of training, goggles were not worn. During PA-i training, a patient wore them the same amount of time. However, in the first series, goggles were donned twice *within* each session and three times in the second series. Pointing movements were kept constant for both conditions and for both phases of prism donning and doffing. Thus, the only difference between PA-i and PA-c was that in the former, patients donned and doffed goggles in-between, whereas patients in the latter conditions wore them without any break.

A total number of at least 50 reaching movements (over the tasks) while wearing the prisms was aimed at for all patients to increase the probability that an after-effect would build up in both conditions. This choice was based on a review by Barrett et al. (29) stating that the direct effect of the optical shift will disappear after about 50 movements. When the patient did not seem to complete the required number of trials before the end of the therapy session, the therapist read out a randomized sequence of numbers in the last 1–2 min of the session, requiring the same movements to the respective buttons as in the prepared stimulus list.

### Procedure

To increase the motivation of the severely impaired patients and to enable an early start of the treatment, a set of five individually adjustable tasks (four computerized tasks and one non-computer-based task) were offered over the two intervention periods. During the computerized tasks, the patient sat centered in front of a computer screen and a RehaCom response panel containing buttons, numbered from 0 to 9 [about 30 cm distance between leftmost and rightmost keys; (36)]. An external button was placed outside a patient's visual field, about 50 cm from the body midline (right-hand side). It served as the starting point for all reaching movements and ensured that a patient could only view the last terminal 10 cm of a movement (after crossing the visual barrier with the hand). A patient was asked to make rapid and visually-guided reaching movements toward the button on the panel corresponding with the target stimulus on the screen. Reaching distance (e.g., more to the left/right) was randomly varied and depended on the required button press (0 = leftmost, 9 = rightmost). The response had to be confirmed by a subsequent movement back to the external button (“OK”) and the hand was finally laid down at that place. All patients followed this procedure both when wearing the goggles and while not wearing them.

The first of five tasks, used to practice with the goggles, was the RehaCom module “Attention and Concentration” (36). Patients were asked to search for a picture (indicated by a number from 1 to 9) that matched the target picture presented continuously during each trial on the right side of the screen. The corresponding button was pressed after finding the picture. The search space was to the left of the target and included several similar pictures and one identical to the target. Task difficulty was automatically raised after 20 correct responses (by default) by changing the number of pictures in the search set (e.g., 3, 6, or 9) or by changing the pictures' richness of details.

The second task was the RehaCom module “Saccadic Training” (36). The patient was first looking at a fixation point in the middle of the screen. During the task, target stimuli appeared on the left and right sides of the screen accompanied by an auditory signal. The finding of the object was indicated by either pressing the left or right arrow key on the keyboard. The stimuli could differ in size, speed of appearance, and contrast with the background. Task difficulty was automatically raised in case of 90% correct responses during each block of 30 items. The optimal reaction time was set to 500 ms (by default).

The third and fourth tasks had been programmed by one of the authors (HH) and had been used in another study (33). In these tasks, clearly visible pop-out stimuli were presented to direct the patient's attention toward the left side of the space. The distribution of stimuli on the left and right sides was biased toward the left (75/25%). More specifically, the third task entailed a reading exercise. Words (spelled-out numbers corresponding to the buttons on the panel, e.g., FOUR = 4) were presented in random order at random points on a virtual horizontal line on the screen. The patient was asked to press the corresponding button after correctly reading the word. The difficulty of spotting a target was adjusted by presenting the word further to the left or right) and by including or excluding additional distracting letters after the word (e.g., FOURTIOA). The fourth task (illustrated in Figure 3) involved searching for a random number that was presented at different points on a horizontal axis on the screen (as in the second task) among a variable number of distractors. To make the task easier, the number could be highlighted, either in bright blue or red. Additionally, the number of distractors, as well as the search space could be adjusted.

The procedure of the non-computerized pegboard task was similar to that of Gossmann et al. (34). A patient was seated right in the middle of the board. The therapists offered sticks, one by one, to the patient who then had to place these into a hole on any position on the pegboard. The task was to place as many sticks as possible for the duration of the therapy block. If all sticks had been placed, the patient was asked to grab the sticks, one by one, and give them back to the therapist. Task difficulty was adjusted by asking the patient to place or grab the stick that was located on the left side of the pegboard.

### Additional Rehabilitative Programs

Apart from the PA treatment, all patients participated in other rehabilitative programs for 5 h daily, commonly offered in an early rehabilitation unit in Germany: physical therapy, occupational therapy as well as computer-based neuropsychological and nursing interventions which, among others, aim at neglect symptom reduction.

### Primary Outcome Measures

#### Apples Cancellation Test

Performance on the Apples Cancellation Test (35) was used as a primary outcome measure. It consists of 150 apples with either a gap on the right- or left-hand side, or no gap (50 apples each), equally distributed over an A4 sheet of paper. Patients were instructed to cross out full apples within 5 min and the number of omitted apples was used as the outcome score.

#### Assessment of Activities of Daily Living

The German version of the Early Rehabilitation Barthel Index [ERBI; (37)] and of the Functional Independence Measure [FIM; (38)] were used to assess difficulties in ADL, with lower scores indicating more care-dependency.

### Secondary Outcome Measures

#### Text Reading

A patient's reading performance was evaluated using four different texts (39), one in each assessment, with a predefined order for each patient. Reading errors (omissions and incorrectly read words) for each text were used for analyses.

#### Line Bisection Test

The Line Bisection Test (40) included three equally long (21 cm) lines, one below the other, with their starting points progressively shifted toward the left. The mean deviation between the marked and the true center was taken as the outcome score.

#### Clock Drawing Test

In the Clock Drawing Test, which is part of the Behavioral Inattention Test (41), an empty circle was presented to the patient, who was asked to fill in the numbers from 1 to 12 in a clockwise direction. The following scoring scheme was applied: 2 points if the clock did not have any number on the left side (“maximally distorted”), 1 point if it showed numbers on both sides but with asymmetrical positioning (“minimally distorted”), and 0 points if the clock did not show spatial distortions (“correct”).

#### Body Orientation

A patient's spontaneous and left-cued body orientation was evaluated separately for trunk, head, and eyes while being in the patient room (42). For the cued body orientation, the evaluator asked three distinct questions centrally in front of the patient: “How are you?,” “Did you sleep well?,” “How was therapy today?” It was scored as follows: zero points for normal position, one point for modest ipsilesional deviation, and two points for strong ipsilesional deviation. The maximal score is six points, with higher scores indicating more ipsilesional deviation of the posture.

### Blinding of Test Evaluation

All measures based on subjective evaluation (body orientation, ERBI, FIM) were scored double-blind by a person other than the one who provided the PA training. The Clock Drawing Test was administered by the therapist but scored by another person. Patients were blind in the sense that they knew about the two training methods but not about underlying assumptions regarding potential differences in their efficiency.

### After-Effect Size

The size of the after-effect was calculated for the period of not wearing the goggles and for the first five pointing movements (in case of PA-i: after patients removed the goggles for the first time). The number of pointing movements not reaching the defined target button and/or pointings that were corrected during movement execution (e.g., accurate movement = 0, left-biased movement, and wrong button pressed = 2) were added. Subsequently, the mean across the sessions was calculated. Higher scores indicated stronger after-effects.

### Statistical Analysis

The following statistical analyses were performed with SPSS Statistics, version 27. Means and standard deviations were calculated. Chi-squared tests and independent *t*-tests, as well as Mann-Whitney *U*-tests, were performed to check for significant group differences in the demographic data and pre-treatment test results. For intra-group comparisons of experimental data, parametric dependent *t*-tests and non-parametric Wilcoxon signed-rank tests were conducted, depending on whether or not the data were normally distributed. Multiple linear and ordinal regression analyses were applied after respective assumption checking (outliers, multicollinearity as well as independence, homoscedasticity, and normal distributions of the residuals) to test if the factor Condition (intermittent, continuous) together with the factors Age, Sex, and PRE significantly predicted POST scores.

Statistical evaluation of possible carry-over effects for the primary and secondary outcome measures included analyses of variance (ANOVAs) for repeated measurements (43). Only patients being able to finish both the first and second conditions of the study were included in the analyses. Time point (PRE vs. POST) was used as the within-subject variable and Sequence (PA-i^1st^ vs. PA-c^1st^) as the between-subject variable. Furthermore, Spearman's rank correlation coefficients were calculated for the after-effect and improvements in outcome parameters.

Apart from the main analysis, we additionally compared the treatment effects of the PA protocols in the current study with those from three other neglect interventions at the early rehabilitation unit of the Clinic Bremen-East. The study by Schenke et al. (32) implemented 15 sessions with passive dynamic auditory cueing (such as music or audio-books). The study by Gossmann et al. (34) offered only four sessions of PA with a grasping task toward a solitaire board. The active control group from Turgut et al. (33) received 15 general neuropsychological therapy sessions not targeting visuospatial abilities but other cognitive domains such as memory and general attention. Clinical characteristics of patients from the present study and those from the three earlier studies were checked for group differences using the Kruskal-Wallis test for continuous variables and Chi-squared test for categorical variables. Subsequently, effect sizes [Cohen's *d*; (44)] were computed by firstly calculating a difference treatment score (POST minus PRE) for every patient (all studies). The following formula was used and implemented in Microsoft Excel:


$$\begin{array}{l} d\;=\;\frac{M_{1}-M_{2}}{SD_{pooled}} \end{array}$$

where *M*_1_ represents the mean of test scores before the intervention and *M*_2_ the mean after the intervention. Here, *SD*_*pooled*_ is the pooled standard deviation for the samples (*n*_1_ = *n*_2_) with


$$\begin{array}{l} SD_{pooled}\;=\;\sqrt{\frac{SD_{1}^{2}\;+\;SD_{2}^{2}}{2}} \end{array}$$

Additionally, the 1 - α (95%) confidence interval (CI) was calculated, using an approach of Hedges and Olkin (45):


$$\begin{array}{l} d\;\pm \text{ se }*\;{\text{z}}_{\text{crit}} \end{array}$$

where *z*_*crit*_ is the critical z value for the level of confidence (1.96 for our 95% CI) and *se* is the standard error of the calculated effect size with


$$\begin{array}{l} \text{se }=\;\sqrt{\frac{{\text{n}}_{1}\;+\;{\text{n}}_{2}}{{\text{n}}_{1}{\text{n}}_{2}}\;+\;\frac{{\text{d}}^{2}}{2\left({\text{n}}_{1}\;+\;{\text{n}}_{2}\right)}} \end{array}$$

Following Cohen (44), a *d* value exceeding 0.2 was considered as a small effect, exceeding 0.5 as a medium, and exceeding 0.8 as a large effect. For the effect size comparison, only the first condition of the cross-over design was used for patients of the present study to guarantee identical time points between measurements and to avoid influences due to an already accomplished intervention.

## Results

### Clinical Characteristics, Test Results, and Pre-treatment Group Differences Between the Two Recruitment Series

In the first series, *n* = 10 patients were included in the PA-i (6 + 4) and *n* = 9 in the PA-c (6 + 3) condition. In the second series, *n* = 9 (5 + 4) were included in the PA-i condition and *n* = 12 (7 + 5) patients in the PA-c condition. There were no significant group differences for the two PA-i and PA-c groups (series one vs. series two) on the primary outcome parameters or the descriptive characteristics. Therefore, we combined the respective conditions for further analyses (compare flowchart in Figure 1; PA-i: *n* = 19, PA-c: *n* = 21). Demographic data and test scores of these two combined groups are presented in Tables 1, 2. There were no significant differences between the groups with regard to age [*t*_(38)_ = 0.29, *p* = 0.776], etiology [$\chi_{\left(2\right)}^{2}$ = 0.02, *p* = 0.99], or the presence of visual field deficits [$\chi_{\left(1\right)}^{2}$ = 0.06, *p* = 0.815] and motor impairment [$\chi_{\left(1\right)}^{2}$ = 0.33, *p* = 0.567]. Furthermore, there were no significant differences in the pre-treatment results on the Apples Cancellation Test (*Z* = −0.52, *p* = 0.611), text reading (*Z* = −0.72, *p* = 0.486), Line Bisection Test [*t*_(38)_ = 0.16, *p* = 0.875], Clock Drawing Test (*Z* = −0.13, *p* = 0.915), spontaneous body orientation (*Z* = −1.10, *p* = 0.283), cued body orientation (*Z* = −0.17, *p* = 0.879), ERBI [*t*_(38)_ = 0.49, *p* = 0.63] or FIM (*Z* = −0.79. *p* = 0.436). The evaluation of possible carry-over effects, i.e., the sequence effect of undergoing PA-i or PA-c first, did not reveal significant differences on any of the primary and secondary outcome measures.

**Table 2:** Test results of the two combined PA groups (both series and after completing both conditions of cross-over design) following an intermittent or continuous protocol.

	**PA-i (*****n*** **= 19)**		**PA-c (*****n*** **= 21)**	
**Test results**	**PRE**	**POST**	**PRE**	**POST**
Apples Cancellation Test (omissions)	28.8 ± 17.5	17.7 ± 17^*^	33.8 ± 13.2	19.9 ± 15.4^*^
Text reading (omissions)	16.6 ± 19.9	8.4 ± 12.6^*^	22.8 ± 20.6	7.2 ± 13^*^
Line Bisection Test (cm)	3.4 ± 3	2.3 ± 1.9	3.6 ± 3.1	2.1 ± 2.3^*^
Clock Drawing Test	0.9 ± 0.8	0.6 ± 0.6^*^	0.9 ± 0.7	0.6 ± 0.6
Spontaneous body orientation	3.1 ± 2.1	1.3 ± 1.2^*^	2.4 ± 1.7	1.9 ± 1.7
Cued body orientation	2 ± 1.7	0.8 ± 1.2^*^	1.9 ± 1.6	1.6 ± 1.8
Early Rehabilitation Barthel Index	−57.6 ± 62.5	16.8 ± 36.8^*^	−48.6 ± 55.5	15.7 ± 36^*^
Functional Independence Measure	47.9 ± 13.1	68.3 ± 19.3^*^	46.5 ± 20.8	63.9 ± 19.7^*^

*Values as means ± standard deviation*.

* *p < 0.05 between pre-and post-treatment measurement*.

*PA, prism adaptation; PA-i, intermittent prism adaptation; PA-c, continuous prism adaptation; PRE, pre-treatment measurement; POST, post-treatment measurement*.

### Effects of the PA Protocols

#### Within-Group Improvements

Intragroup comparisons showed significant improvements from PRE to POST for the PA-i group for omissions on the Apples Cancellation Test [*t*_(18)_ = 3.97, *p* = 0.001], Clock Drawing Test (*Z* = −2.27, *p* = 0.023), text reading [*t*_(18)_ = 2.16, *p* = 0.044], spontaneous body orientation (*Z* = −3.2, *p* = 0.001), cued body orientation [*t*_(17)_ = 2.58, *p* = 0.019], ERBI (*Z* = −3.4 *p* = 0.001) as well as the FIM (*Z* = −3.7, *p* < 0.001). However, the PA-i group did not improve on the Line Bisection Test [*t*_(18)_ = 1.73, *p* = 0.101]. Patients in the continuous PA training condition improved significantly on the Apples Cancellation Test [*t*_(20)_ = 4.19, *p* < 0.001], Line Bisection Test (*Z* = −2.36, *p* = 0.018), text reading (*Z* = −3.24, *p* = 0.001), ERBI [*t*_(20)_ = −5.51, *p* < 0.001], and FIM [*t*_(20)_ = −7.5, *p* < 0.001]. However, the PA-c group did not improve on the Clock Drawing Test (*Z* = −1.83, *p* = 0.067) or spontaneous [*t*_(19)_ = 1.34, *p* = 0.196] and cued body orientation (*Z* = −0.56, *p* = 0.573).

#### Regression Analyses

Multiple linear and ordinal regression analyses with Condition (intermittent, continuous), Age, Sex, and PRE as independent variables (predictors), and POST as the dependent variable were conducted (see Tables 3, 4). It was found that Condition did not significantly predict scores on any of the primary outcome parameters. For secondary outcome measures, we found a significant effect on spontaneous body orientation only: Condition [Wald $\chi_{\left(1\right)}^{2}$ = 4.23, *p* = 0.04] and PRE [Wald $\chi_{\left(1\right)}^{2}$ = 12.75, *p* < 0.001] significantly predicted POST (pseudo $R_{\text{Nagelkerke}}^{2}$ = 41.1%]. The odds of patients in the PA-i having a better spontaneous body orientation was 4.27 (95% CI, 1.07–17.01) times that of those in the PA-c condition.

**Table 3:** Ordinal linear regression models examining post-treatment neuropsychological test results (*N* = 40).

**Variables**	***b***	**SE**	***p***	**95% CI**	
				**Lower**	**Upper**
**Dependent variable: Clock Drawing Test (POST)**					
Age	0.04	0.03	0.10	−0.01	0.10
Sex	−0.13	0.63	0.83	−1.36	1.10
PRE	2.30	0.55	<0.001^*^	1.22	3.39
Condition	0.54	0.63	0.39	−0.69	1.76
**Dependent variable: spontaneous body orientation (POST)**					
Age	0.02	0.03	0.35	−0.03	0.07
Sex	0.05	0.64	0.94	−1.21	1.30
PRE	0.77	0.22	<0.001^*^	0.35	1.19
Condition	1.45	0.71	0.04^*^	0.07	2.83
**Dependent variable: cued body orientation (POST)**					
Age	0.02	0.02	0.93	−0.04	0.05
Sex	0.10	0.62	0.87	−1.11	1.32
PRE	0.34	0.19	0.08	−0.04	0.05
Condition	0.96	0.63	0.13	−0.27	2.20

* *p < 0.05*.

*PRE, pre-treatment measurement; POST, post-treatment measurement*.

**Table 4:** Multiple linear regression models examining post-treatment neuropsychological test results (*N* = 40).

**Variables**	***b***	**SE**	**β**	***t***	***p***
**Dependent variable: Apples Cancellation Task (POST)**					
Age	0.21	0.17	0.16	1.22	0.23
Sex	−2.30	4.12	−0.07	−0.56	0.58
PRE	0.69	0.14	0.66	4.93	<0.001^*^
Condition	1.50	4.17	0.05	0.36	0.72
**Dependent variable: text reading (POST)**					
Age	0.02	0.15	0.02	0.15	0.89
Sex	−0.83	3.66	−0.03	−0.23	0.82
PRE	0.33	0.09	0.53	3.60	0.001^*^
Condition	3.22	3.66	0.13	0.88	0.39
**Dependent variable: Line Bisection Test (POST)**					
Age	0.01	0.03	0.06	0.39	0.70
Sex	−0.13	0.63	−0.03	−0.20	0.84
PRE	0.34	0.11	0.49	3.14	0.003^*^
Condition	0.25	0.60	0.06	0.42	0.68
**Dependent variable: Early Rehabilitation Barthel Index (POST)**					
Age	−0.34	0.46	−0.12	−0.73	0.47
Sex	13.06	11.40	0.18	1.15	0.26
PRE	0.15	0.10	0.24	1.48	0.15
Condition	2.04	11.38	0.03	0.18	0.86
**Dependent variable: Functional Independence Measure (POST)**					
Age	−0.35	0.20	−0.22	−1.74	0.09
Sex	1.16	4.92	0.03	0.24	0.82
PRE	0.68	0.14	0.61	4.76	<0.001^*^
Condition	3.07	4.92	0.08	0.62	0.54

* *p < 0.05*.

*PRE, pre-treatment measurement; POST, post-treatment measurement*.

### After-Effect Size

For the PA-i condition, the mean number of reaching movements toward the keyboard was 97.09 [*SD* = 56.29; range = (31.40, 218.47)] while wearing the goggles and 41.18 [*SD* = 21.4; range = (20.80, 108.80)] when not wearing them (total: *M* = 138.28, *SD* = 74.22). For PA-c these values were 105.63 [*SD* = 58.76; range = (48.07, 219.27)] and 31.44 [*SD* = 10.01; range = (12.13, 51.20)] (total: *M* = 137.07, *SD* = 64.23). Only in the first phase of the study (intervention 1), there was one patient in each condition who did not reach the criterion of 50 pointing movements per session while wearing the goggles.

**Figure 4:**
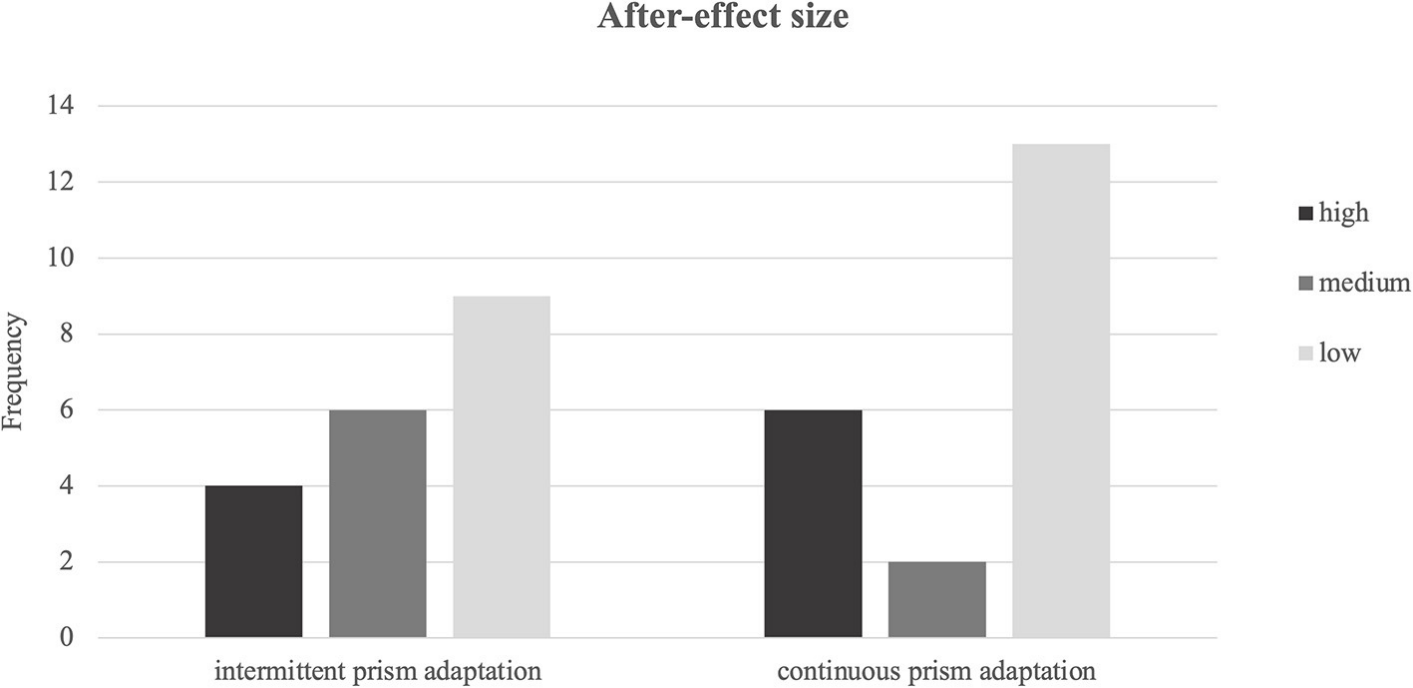
Frequencies of after-effect sizes for intermittent and continuous prism adaptation training categorized into low, medium, and high.

All patients showed an after-effect (size > 0) and there was no difference between the two groups (*Z* = −0.37, *p* = 0.714). For a further comparison across the two PA protocols, we classified the after-effect size into low (lowest third of values), medium (middle third), and high (highest third) (see Figure 4). Most patients showed a small after-effect in both the PA-i and PA-c conditions. In the PA-i condition, more patients showed a medium rather than a low after-effect, whereas it was vice versa for the PA-c patients. Overall, PA-i and PA-c did not significantly differ regarding the three categories (low, medium, and high) as revealed by a Chi-squared test [$\chi_{\left(2\right)}^{2}$ = 3.04, *p* = 0.219]. Furthermore, there was no significant difference in the total number of reaching movements (*Z* = −0.12, *p* = 0.903). The size of the after-effect did not correlate with improvements on any of the primary outcome parameters. However, a significant positive correlation was found between after-effect size (both PA-i and PA-c groups together) and improvements of spontaneous body orientation (*r* = 0.34, *p* = 0.037).

### Analysis of Drop-Outs After the First Intervention

In order to analyze the patient profiles of those who dropped out after the first intervention (*n* = 8; *n* = 5 when PA-c was given first, *n* = 3 when PA-i was given first), we checked for significant differences compared to those who successfully crossed-over to the second intervention (*n* = 16). We did not find any significant differences in patient characteristics or neuropsychological test scores at T3. However, they significantly differed in ERBI scores (*Z* = −3.05, *p* = 0.001), with drop-outs having lower scores (*M* = −3.44, *SD* = 29.42) than patients who started the second intervention (*M* = 43.13, *SD* = 27.77).

### Effect Size Comparisons With Prior Studies

**Figure 5:**
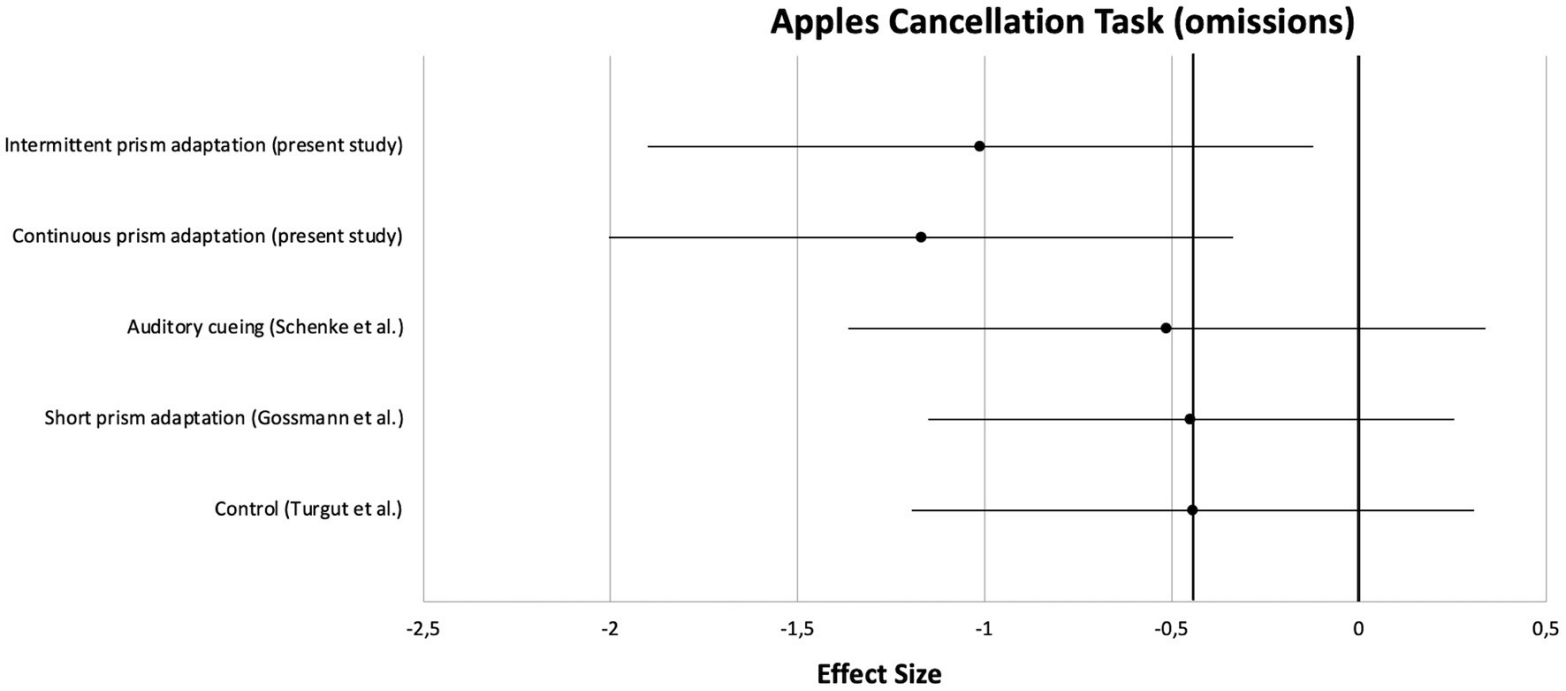
Comparison of effect sizes of the present prism adaptation treatment protocols and previous studies on neglect interventions for the Apples Cancellation Test.

**Figure 6:**
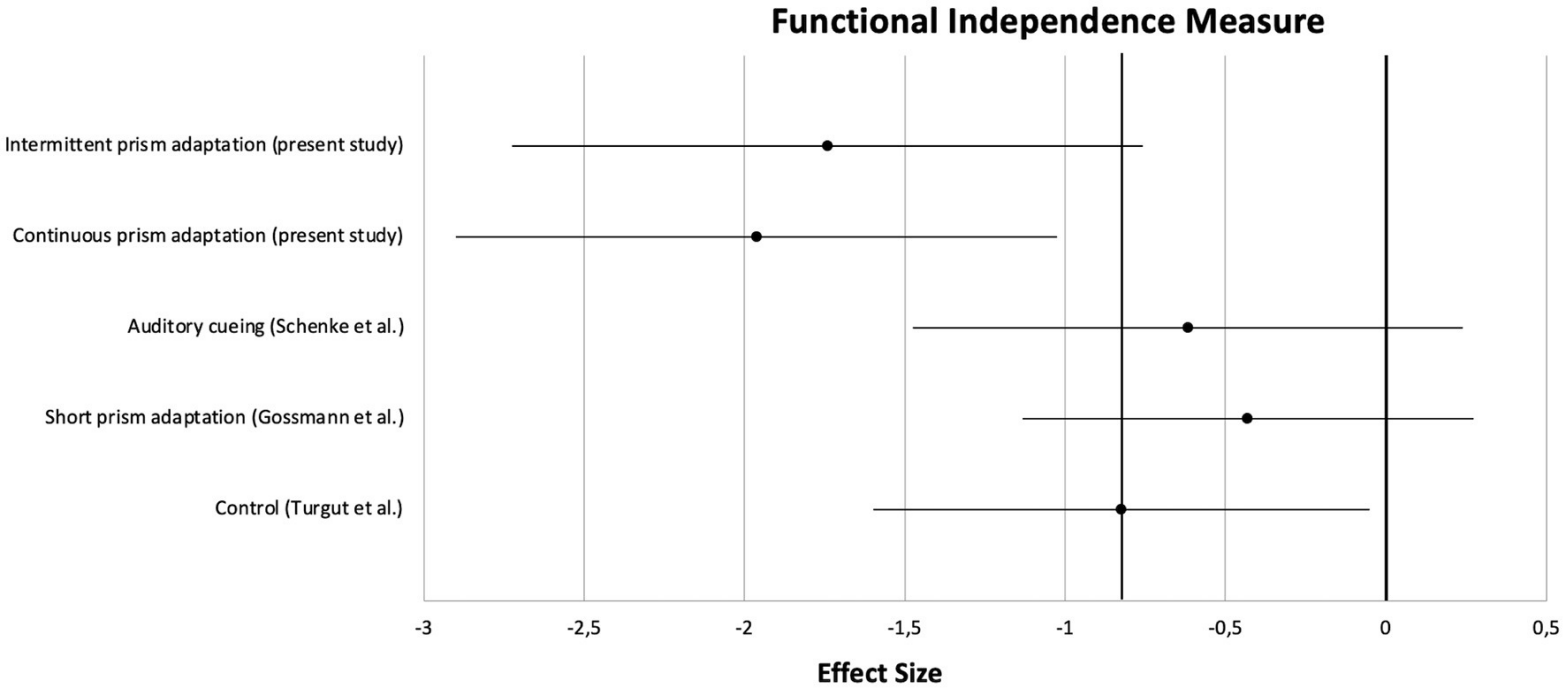
Comparison of effect sizes of the present prism adaptation treatment protocols and previous studies on neglect interventions for the Functional Independence Measure.

Apart from the main analysis, effect sizes of pre- to post-treatment improvements were calculated and compared with those from the three previous studies. Clinical characteristics of the patients are summarized in Table 5. For a comparison on a central neuropsychological task, the Apples Cancellation Test, see Figure 5. For the double-blind evaluated ADL assessment FIM, see Figure 6.

**Table 5:** Clinical characteristics of the present prism adaptation treatment protocols (first condition only) and previous studies on neglect interventions.

	**PA-i**	**PA-c**	**Schenke et al**.	**Gossmann et al**.	**Turgut et al**.
**Treatment**	PA	PA	Auditory cueing	PA	Placebo
**Session duration (min)**	30–40	30–40	30	30	20–40
**Clinical characteristics**					
*N*	11	13	11	16	14
Sex (F:M)	5:6	6:7	3:8	7:9	4:10
Age (years)	61.2 ± 14.5	61.4 ± 13.1	69.2 ± 10.1	69.3 ± 9.9	67 ± 14
Days since stroke	21.1 ± 15.5	21.6 ± 19.6	35.4 ± 46.7	36.3 ± 19.9	42.1 ± 36.8
**Etiology**					
Ischemia	4	7	6	15	12
Intracerebral hemorrhage	6	4	4	1	1
Subarachnoid hemorrhage	2	2	1	0	1
Mixed	1	2	1	0	1
**Test results (baseline)**					
Apples Cancellation Task (omissions)	34.2 ± 16.3	37.0 ± 11.7	41.5 ± 10.2	39.3 ± 10.6	44.0 ± 8.2
Functional Independence Measure	40.5 ± 7.6	35.9 ± 8.6	47.9 ± 20.4	33.2 ± 9.8	36.4 ± 13.5

*Values as means ± standard deviation for continuous variables*.

*PA, prism adaptation; PA-i, intermittent prism adaptation; PA-c, continuous prism adaptation*.

Patients from the two PA intervention protocols of the present study and those from the previous studies did not significantly differ with regard to age [*H*_(4)_ = 5.97, *p* = 0.202], days since stroke [*H*_(4)_ = 7.62, *p* = 0.106], sex [$\chi_{\left(4\right)}^{2}$ = 1.9, *p* = 0.754] or pre-treatment results on the Apples Cancellation Test [*H*_(4)_ = 8.27, *p* = 0.082], and FIM [*H*_(4)_ = 6.29, *p* = 0.179] (see Table 5).

On the Apples Cancellation Test, patients treated with auditory cueing or a short PA intervention showed similar effect sizes (*d* = −0.51 and *d* = −0.45, respectively) as the control group receiving no specific neglect training (*d* = −0.44). However, as can be seen in the Figure, the effect sizes of the two PA groups from the present study were clearly higher (at least more than half an *SD*; PA-i: *d* = −1.01, PA-c: *d* = −1.17). A similar pattern was observed for the FIM: whereas PA-i and PA-c improved almost two *SD*s (*d* = −1.75 and *d* = −1.96, respectively), the passive control groups improved below 1 *SD* each.

## Discussion

The present study investigated whether recalibration or realignment is more important for the reduction of neglect severity by offering either a conventional continuous PA training or a novel intermittent PA training that offers additional recalibration opportunities and, hence, for using explicit strategies [such as pointing slightly left of the perceived target location; (8)] more frequently.

Patients in both conditions improved significantly on all primary as well as many secondary outcome measures. However, treatment condition *per se* did not significantly predict scores on primary and most secondary outcome parameters. Therefore, neither the higher number of opportunities for recalibration (intermittent PA) nor the duration of uninterrupted wearing of the goggles (continuous PA) had a differential effect on recovery from neglect as measured by a visual search task or by ADL scores.

What might explain this disappointing result of our study? A first answer would be that PA is ineffective in treating neglect. Two recent RCTs including patients with mild (46) or severe (47) neglect revealed negative results, arguing against a positive effect of PA on neglect recovery. However, other studies documented positive results for PA (34, 48–51), and we will argue below that both our PA conditions were effective compared to previous neglect treatments evaluated at our rehabilitation unit. A second answer relates to our finding that there was no significant difference between the PA-c and PA-i conditions in inducing an after-effect of considerable size. It is well-known that the size of the after-effect may represent an important measure in reflecting the efficacy of PA (27, 52–54), although there have been described cases showing an after-effect but no improvements or vice versa (55, 56). Because both conditions showed a similar after-effect size, there might have been no differential effects on neglect rehabilitation. We designed our study in order to induce comparable sizes of the after-effect between the two conditions. We opted for a minimum criterion of 50 pointing movements per session [(29); patients clearly executed more pointings than this criterion; see section *After-Effect Size*] in order to increase the probability that adaptation (and thus, an after-effect) will occur (30). The reason for defining this criterion was that we wanted to show whether explicit recalibration or implicit realignment is the driving force for recovery after PA. Hence, we wanted to avoid a confounding influence of an after-effect difference between conditions because such a difference itself could explain the superiority of one treatment protocol over another (53).

Failing on our primary outcome parameters, the two PA conditions still showed a difference in efficacy when looking at rebalancing *body orientation*, which is an essential aspect of ADL, and its evaluation is strongly advised in the German neurological guidelines for deficits in spatial cognition (57). This finding is in line with previous research showing that body adaptation (eyes, head, and trunk) occurred after PA, even irrespective of pointing movements (58). Patients in the intermittent condition needed to recalibrate more frequently (at least each time immediately after putting on the goggles) and showed the after-effect more often than patients in the PA-c condition (each time immediately after putting off the goggles). Moreover, we found the size of the after-effect to significantly correlate with improvements in ipsilesional spontaneous body orientation. Therefore, in our study, the after-effect in general seemed to represent an essential sign of improvements in body orientation. This is, as outlined above, in line with previous studies showing that the after-effect is associated with and might predict neglect amelioration (27, 52–54).

Somewhat speculatively, it could be proposed that the *number* (after the doffing of the goggle) and *intensity* of the experienced after-effects lead to a rebalancing of body orientation. If this is true, the question remains why the after-effect together with the reduction of pointing errors (i.e., employing recalibration) following PA should actually be that important. One explanation might be that a change in spontaneous body posturing after PA might be remembered implicitly by the patients as helpful for task accomplishment, which could result in immediate body position adjustments when goggles are put on again. This suggestion is at least in line with results from a study by Pochopien and Fahle (31) who investigated how the direct effect (i.e., initial pointing error when goggles are put on, which is equivalent to the start of the recalibration process) of PA is corrected in healthy adults. In an experimental study, they compared participants sitting with their body orthogonal to the visual target with those whose chair was rotated to the right (more or less comparable to the body orientation of neglect patients). They found that the initial correction effect (that is, the discrepancy between the degree of induced visual shift by the prism and the much smaller than expected degree of initial pointing error) was significantly larger for central rather than rotated chair positions. In other words, pointings deviated less from the actual position of the target when the body was oriented in line with the visual target. Considering neglect patients, slightly rotating the body contralaterally might help to reduce their pointing errors and this might be learned better in a PA-i training, possibly due to two reasons: (1) patients in the PA-i condition undergo changes of body orientation more frequently and (2) these patients have much shorter time intervals between experiences of helpful body orientations (a few minutes within the session) as opposed to patients in the PA-c condition (a whole day between the sessions). These aspects could be advantageous for the intermittent condition and might explain our findings.

At this point it should be noted again that our study overall failed to show beneficial effects of one protocol over the other due to the lack of difference in primary outcome parameters. Our study was designed to compare two PA conditions and not to show that PA is effective for treating neglect. However, since several studies on neglect rehabilitation have been conducted in our rehabilitation unit over the last years, we were able to calculate and compare effect sizes of “recovery” for three groups from already published studies with those obtained by the present study. We chose the Apples Cancellation Test as a central neuropsychological measure and the Functional Independence Measure as an ADL score for the comparisons because the Apples Cancellation Test is well-validated (59), seems to replace older cancellation tasks (60), is easy to score, and scoring itself is not influenced by a subjective bias. The Functional Independence Measure is even less influenced by this issue because evaluators (hospital staff such as medical doctors, occupational and physiotherapists as well as nurses who routinely score every in-patient weekly) were not aware of a patient's specific inclusion into the studies at all. Effect sizes appeared to be strongest for both PA treatment protocols when being compared to the historical control groups. The difference between the most successful treatment of the historical control group and the two PA protocols used in our study was about half a *SD* for the Apples Cancellation Test and one *SD* for the FIM. According to Cohen, these differences can be classified as medium and large effects, respectively (44). We found that active neglect treatment appeared to be more effective than a passive treatment (hearing audiobooks or CDs with auditory shift), a more intensive PA training revealed to be more effective than a much shorter PA training (albeit with different tasks), and the effects of our PA protocols appeared to be distinguishable from effects due to only spontaneous recovery (active control group).

As already mentioned, two recent RCTs on PA were unable to show a large impact on neglect rehabilitation (46, 47). In contrast, our comparison with three historical control groups does argue for such an effect. What may explain this difference? Neglect patients often suffer not only from visuospatial attention deficits, but also from apathy, unawareness, and reduced motivation. Traditional PA interventions are based on rather meaningless pointing movements toward the center of a line or different positions in the environment. This task structure may even increase the possible lack of involvement of neglect patients. Therefore, we combined PA into several meaningful tasks, also rewarding correcting choices, and we increased difficulty based on the performance of the patients. In our view, this is an important difference from the designs of Vilimovsky et al. or Ten Brink et al., and we believe that this kind of adaptation of PA is essential for treating especially severely impaired patients with neglect due to their additional emotional and motivational impairments. This claim is supported by previous studies on reward-based learning following right hemisphere damage and neglect, showing that reward-induced modulations of space representation are preserved in neglect (61), reward positively influences spatial exploration (62), motivational valence modulates attentional impairments (63), and reward-based training may benefit most patients early after lesion onset (62).

Eight out of 24 recruited patients did not (completely) receive the second intervention due to rehabilitation discharge. Comparisons of these patients with those who crossed-over to the second PA treatment revealed no significant differences in clinical characteristics or neuropsychological test results. Thus, drop-outs seem not to have certain disease profiles in our study. Only the ERBI differed significantly, indicating that patients were most likely discharged to their home or for participation in the next rehabilitation phase (in a subsequent rehabilitation clinic).

A few limitations of this study warrant consideration. First, effects due to selection bias cannot be excluded entirely. This is not only because patients included in our main analyses were recruited within two recruitment series but also because the historical control groups included for a general treatment effect evaluation were treated at different moments. However, they were all recruited for neglect intervention studies and the latter group already showed significant improvements following neglect therapy, which has been published. Moreover, all patients, also those from the three control groups, were recruited from the same rehabilitation unit, with similar selection criteria; they matched in terms of the choice and time course of neuropsychological and ADL assessments and received the same additional rehabilitative interventions. Although tasks being used in the historical groups differed from those implemented in the present study, all have in common that they combine spatial cueing with meaningful tasks, except for the passive control group. As noted earlier, we chose for such a combination treatment to enable patients to participate in therapy as early as possible and not primarily to induce add-on effects [these have been shown to be very low in previous studies, e.g., (64)]. Therefore, the comparison appears to be fair and relevant.

Second, we did not assess the size of the after-effect in a conventional way (e.g., pre-and post-exposure straight-ahead pointing in the dark or video analysis). There is some evidence that the size may depend on the specific measure used (54). We aimed to make PA training sessions as feasible as possible for severely impaired early rehabilitation patients and the advantage of our assessment method was that it could be easily implemented *within* the actual treatment session. Our rather subjective and less sensitive measure might have missed subtle differences in sizes of the after-effect, but for our study, the more important fact is that patients of both groups showed the effect and also showed some recovery.

Third, we only measured the treatment effect 1 day after the PA training, preventing us from drawing inferences about long-term effects of these interventions (i.e., stability of neglect symptoms). The nature of our design included seven weeks of patient involvement, which is often the maximum stay of inpatients in a German early rehabilitation unit. Hence, an evaluation of long-term effects of PA would only be possible in subsequent rehabilitation or care units as well as at home. Here, comparability would be reduced due to different contexts and varying amounts of therapy offered.

Fourth, although we provide global information on lesion locations, exact information is lacking. From previous studies it is known that lesion location can influence PA treatment response of neglect patients (34, 62, 65). However, most of the patients (16 out of 24) received both phases of the cross-over and, hence, both interventions, minimizing possible effects solely due to specific lesion characteristics. Nevertheless, future studies should include and control for this missing information in order to validate the findings of the present work.

Lastly, we did not implement PA protocols that induced recalibration *without* realignment or vice versa. Thus, in the PA-i training, realignment is not precluded. Nevertheless, patients in the PA-i group were still given more opportunities to recalibrate, while keeping the number of movements and the treatment time with goggles on and off constant over the protocols. Furthermore, it is known that the number of pointings is correlated with the intensity of realignment as indicated by stronger after-effects (30, 31). Hence, the two groups in our study did differ with regard to the degree of realignment and the frequency of recalibration and realignment alternations (see above for a discussion on why the after-effect size presumably did not differ between PA-i and PA-c). Nevertheless, especially a protocol inducing only recalibration would be necessary to prove that a stronger after-effect might indeed cause stronger recalibrations, which in turn will lead to greater improvements in spontaneous body orientation. To test this hypothesis, a protocol could include PA training in which goggles are donned only for a short amount of time (e.g., only up to 20 pointings) to avoid realignment that seems to be particularly associated with later trials (8). Patients still showing significant improvements concerning body orientation would provide further evidence and confirm our results. A protocol inducing realignment without recalibration may be achieved by progressively increasing the visual shift from zero to full visual shift, for instance, in steps of two degrees (14, 23). The rationale is that for this procedure the direct effect will always lay in the normal error range of pointing movements.

In conclusion, we found no evidence for an impact of intermittent PA treatment on a variety of typical neglect outcomes, with the exception of one beneficial effect on spontaneous body orientation. preliminary evidence that an intermittent PA procedure may have a beneficial effect on the reduction of a patient's spontaneous body orientation but not on other ADL elements or more perceptual aspects of the disorder. Because we specifically designed our study to induce comparable after-effects for both PA conditions, we suggest that future studies might use fewer pointing movements in PA-i to see if pure recalibration is already effective for achieving improvements in body orientation.

## Data Availability Statement

The raw data supporting the conclusions of this article will be made available by the authors, without undue reservation.

## Ethics Statement

The studies involving human participants were reviewed and approved by Bremen Medical Board's Ethics Committee. The patients/participants provided their written informed consent to participate in this study.

## Author Contributions

JS, SK, AK, and HH conceptualized the study. HH was responsible for the methodology and supervised the study. JS and SK did the investigations. JS and HH performed the statistical analysis. JS, SK, PE, AK, and HH wrote the first draft of the manuscript. JS, PE, and HH reviewed and edited the manuscript. All authors contributed to the article and approved the submitted version.

## Conflict of Interest

The authors declare that the research was conducted in the absence of any commercial or financial relationships that could be construed as a potential conflict of interest.

## Publisher's Note

All claims expressed in this article are solely those of the authors and do not necessarily represent those of their affiliated organizations, or those of the publisher, the editors and the reviewers. Any product that may be evaluated in this article, or claim that may be made by its manufacturer, is not guaranteed or endorsed by the publisher.

## References

[1] Heilman KM Watson RT Valenstein E. Neglect and Related Disorders. Oxford: Oxford University Press (2003). 10.1016/B0-12-226870-9/00804-2

[2] Karnath HO Milner D Vallar G. The Cognitive and Neural Bases of Spatial Neglect. Oxford: Oxford University Press (2002). 10.1093/acprof:oso/9780198508335.001.0001

[3] Kerkhoff G. Spatial hemineglect in humans. Progr Neurobiol. (2001) 63:1–27. 10.1016/S0301-0082(00)00028-9 11040416

[4] Kerkhoff G Schenk T. Rehabilitation of neglect: an update. Neuropsychologia. (2012) 50:1072–9. 10.1016/j.neuropsychologia.2012.01.024 22306520

[5] Gammeri R Iacono C Ricci R Salatino A. Unilateral spatial neglect after stroke: current insights. Neuropsychiatr Dis Treat. (2020) 16:131–52. 10.2147/NDT.S171461 32021206 PMC6959493

[6] Rossetti Y Rode G Pisella L Farné A Li L Boisson D. Prism adaptation to a rightward optical deviation rehabilitates left hemispatial neglect. Nature. (1998) 395:166–9. 10.1038/25988 9744273

[7] Goedert KM Chen P Foundas AL Barrett AM. Frontal lesions predict response to prism adaptation treatment in spatial neglect: a randomised controlled study. Neuropsychol Rehabil. (2018) 30:32–53. 10.1080/09602011.2018.1448287 29558241 PMC6148387

[8] Panico F Rossetti Y Trojano L. On the mechanisms underlying Prism Adaptation: a review of neuro-imaging and neuro-stimulation studies. Cortex. (2020) 123:57–71. 10.1016/j.cortex.2019.10.003 31759324

[9] Redding GM Rossetti Y Wallace B. Applications of prism adaptation: a tutorial in theory and method. Neurosci Biobehav Rev. (2005) 29:431–44. 10.1016/j.neubiorev.2004.12.004 15820548

[10] Redding GM Wallace B. Adaptive Spatial Alignment. Erlbaum: Psychology Press (1997). 10.1037//0096-1523.22.2.379 8934851

[11] Redding GM Wallace B. Prism adaptation during target pointing from visible and nonvisible starting locations. J Mot Behav. (1997) 29:119–30. 10.1080/00222899709600827 12453789

[12] Redding GM Wallace B. Strategie calibration and spatial alignment: a model from prism adaptation. J Mot Behav. (2002) 34:126–38. 10.1080/00222890209601935 12057886

[13] Làdavas E Bonifazi S Catena L Serino A. Neglect rehabilitation by prism adaptation: different procedures have different impacts. Neuropsychologia. (2011) 49:1136–45. 10.1016/j.neuropsychologia.2011.01.044 21310165

[14] Redding GM Wallace B. Prism adaptation and unilateral neglect: review and analysis. Neuropsychologia. (2006) 44:1–20. 10.1016/j.neuropsychologia.2005.04.009 15907951

[15] O'Shea J Revol P Cousijn H Near J Stagg C Rode G. IS 45. Brain stimulation-enhanced therapy for visual neglect. Clin Neurophysiol. (2013) 124:e53. 10.1016/j.clinph.2013.04.064

[16] Rossetti Y Koga K Mano T. Prismatic displacement of vision induces transient changes in the timing of eye-hand coordination. Percept Psychophys. (1993) 54:355–64. 10.3758/BF03205270 8414894

[17] Jeannerod M Rossetti Y. Visuomotor coordination as a dissociable visual function: experimental and clinical evidence. Baillieres Clin Neurol. (1993) 2:439–60. 8137008

[18] Prablanc C Panico F Fleury L Pisella L Nijboer T Kitazawa S. Adapting terminology: clarifying prism adaptation vocabulary, concepts, and methods. Neurosci Res. (2019) 3:3. 10.1016/j.neures.2019.03.003 30910735

[19] Weiner MJ Hallett M Funkenstein HH. Adaptation to lateral displacement of vision in patients with lesions of the central nervous system. Neurology. (1983) 33:766–766. 10.1212/WNL.33.6.766 6682520

[20] Aimola L Rogers G Kerkhoff G Smith DT Schenk T. Visuomotor adaptation is impaired in patients with unilateral neglect. Neuropsychologia. (2012) 50:1158–63. 10.1016/j.neuropsychologia.2011.09.029 21964198

[21] Hanajima R Shadmehr R Ohminami S Tsutsumi R Shirota Y Shimizu T. Modulation of error-sensitivity during a prism adaptation task in people with cerebellar degeneration. J Neurophysiol. (2015) 114:2460–71. 10.1152/jn.00145.2015 26311179 PMC4620141

[22] Hatada Y Miall RC Rossetti Y. Two waves of a long-lasting aftereffect of prism adaptation measured over 7 days. Exp Brain Res. (2006) 169:417–26. 10.1007/s00221-005-0159-y 16328305

[23] Michel C Pisella L Prablanc C Rode G Rossetti Y. Enhancing visuomotor adaptation by reducing error signals: single-step (aware) vs. multiple-step (unaware) exposure to wedge prisms. J Cogn Neurosci. (2007) 19:341–50. 10.1162/jocn.2007.19.2.341 17280521

[24] Petitet P O'Reilly JX O'Shea J. Towards a neuro-computational account of prism adaptation. Neuropsychologia. (2018) 115:188–203. 10.1016/j.neuropsychologia.2017.12.021 29248498 PMC6018603

[25] Panico F Fleury L Trojano L Rossetti Y. Prism adaptation in M1. J Cogn Neurosci. (2021) 33:563–73. 10.1162/jocn_a_01668 33378244

[26] Redding GM Wallace B. Calibration and alignment are separable: evidence from prism adaptation. J Mot Behav. (2001) 33:401–12. 10.1080/00222890109601923 11734414

[27] Striemer CL Russell K Nath P. Prism adaptation magnitude has differential influences on perceptual vs. manual responses. Exp Brain Res. (2016) 234:2761–72. 10.1007/s00221-016-4678-5 27206500

[28] Striemer CL Danckert JA. Through a prism darkly: re-evaluating prisms and neglect. Trends Cogn Sci. (2010) 14:308–16. 10.1016/j.tics.2010.04.001 20444640

[29] Barrett AM Goedert KM Basso JC. Prism adaptation for spatial neglect after stroke: translational practice gaps. Nat Rev Neurol. (2012) 8:567–77. 10.1038/nrneurol.2012.170 22926312 PMC3566983

[30] Fernández-Ruiz J Diaz R. Prism adaptation and aftereffect: specifying the properties of a procedural memory system. Learn Mem. (1999) 6:47–53. 10355523 PMC311278

[31] Pochopien K Fahle M. How to get the full prism effect. Iperception. (2015) 6:1–10. 10.1177/2041669515599308 27433319 PMC4934650

[32] Schenke N Franke R Puschmann S Turgut N Kastrup A Thiel CM. Can auditory cues improve visuo-spatial neglect? Results of two pilot studies. Neuropsychol Rehabil. (2020) 31:710–30. 10.1080/09602011.2020.1727931 32102605

[33] Turgut N Möller L Dengler K Steinberg K Sprenger A Eling P. Adaptive cueing treatment of neglect in stroke patients leads to improvements in activities of daily living: a randomized controlled, crossover trial. Neurorehabil Neural Repair. (2018) 32:988–98. 10.1177/1545968318807054 30328767

[34] Gossmann A Kastrup A Kerkhoff G López-Herrero C Hildebrandt H. Prism adaptation improves ego-centered but not allocentric neglect in early rehabilitation patients. Neurorehabil Neural Repair. (2013) 27:534–41. 10.1177/1545968313478489 23471178

[35] Bickerton WL Samson D Williamson J Humphreys GW. Separating forms of neglect using the Apples Test: validation and functional prediction in chronic and acute stroke. Neuropsychology. (2011) 25:567–80. 10.1037/a0023501 21574718

[36] HASOMED. RehaCom - Cognitive Therapy. (1986). Available online at: https://hasomed.de (accessed June 23, 2021).

[37] Schönle PW. The early rehabilitation barthel index - an early rehabilitation oriented extension of the barthel index. Rehabilitation. (1995) 34:69–73. 7624593

[38] Keith RA Granger CV Hamilton BB Sherwin FS. The Functional Independence Measure: a new tool for rehabilitation. Adv Clin Rehabil. (1987) 1:6–18. 3503663

[39] Wilson B Cockburn J Baddeley A. The Rivermead Behavioural Memory Test Manual. London: Thames Valley Test Corporation (1991).

[40] Schenkenberg T Bradford DC Ajax ET. Line bisection and unilateral visual neglect in patients with neurologic impairment. Neurology. (1980) 30:509. 10.1212/WNL.30.5.509 7189256

[41] Wilson B Cockburn J Halligan P. Development of a behavioral test of visuospatial neglect. Arch Phys Med Rehabil. (1987) 68:98–102. 3813864

[42] Karnath HO Dieterich M. Spatial neglect - a vestibular disorder? Brain. (2006) 129:293–305. 10.1093/brain/awh698 16371409

[43] Glass GV Peckham PD Sanders JR. Consequences of failure to meet assumptions underlying the fixed effects analyses of variance and covariance. Rev Educ Res. (1972) 42:237–88. 10.3102/00346543042003237

[44] Cohen J. Statistical Power Analysis for the Behavioral Sciences. Mahwah, NJ: Erlbaum (1988).

[45] Hedges LV Olkin I. Statistical Methods for Meta-Analysis. Cambridge, MA: Academic Press (1985).

[46] Ten Brink AF Visser-Meily JMA Schut MJ Kouwenhoven M Eijsackers ALH Nijboer TCW. Prism adaptation in rehabilitation? no additional effects of prism adaptation on neglect recovery in the subacute phase poststroke: a randomized controlled trial. Neurorehabil Neural Repair. (2017) 31:1017–28. 10.1177/1545968317744277 29192535

[47] Vilimovsky T Chen P Hoidekrova K Petioky J Harsa P. Prism adaptation treatment to address spatial neglect in an intensive rehabilitation program: a randomized pilot and feasibility trial. PLoS ONE. (2021) 16:e0245425. 10.1371/journal.pone.0245425 33481828 PMC7822563

[48] Abbruzzese L Damora A Antonucci G Zoccolotti P Mancuso M. Effects of prism adaptation on reference systems for extrapersonal space in neglect patients. Brain Sci. (2019) 9:327. 10.3390/brainsci9110327 31744104 PMC6896101

[49] Goedert KM Zhang JY Barrett AM. Prism adaptation and spatial neglect: the need for dose-finding studies. Front Hum Neurosci. (2015) 243:1–7. 10.3389/fnhum.2015.00243 25983688 PMC4415396

[50] Mizuno K Tsuji T Takebayashi T Fujiwara T Hase K Liu M. Prism adaptation therapy enhances rehabilitation of stroke patients with unilateral spatial neglect. Neurorehabil Neural Repair. (2011) 25:711–20. 10.1177/1545968311407516 21700922

[51] Nys GM de Haan EH Kunnenman A de Kort PL Dijkerman HC. Acute neglect rehabilitation using repetitive prism adaptation: a randomized placebo-controlled trial. Restor Neurol Neurosci. (2008) 26:1–12. 18431002

[52] Farnè A Rossetti Y Toniolo S Làdavas E. Ameliorating neglect with prism adaptation: visuo-manual and visuo-verbal measures. Neuropsychologia. (2002) 40:718–29. 10.1016/S0028-3932(01)00186-5 11900724

[53] Gutierrez-Herrera M Eger S Keller I Hermsdörfer J Saevarsson S. Neuroanatomical and behavioural factors associated with the effectiveness of two weekly sessions of prism adaptation in the treatment of unilateral neglect. Neuropsychol Rehabil. (2018) 30:187–206. 10.1080/09602011.2018.1454329 29860929

[54] Sarri M Greenwood R Kalra L Papps B Husain M Driver J. Prism adaptation aftereffects in stroke patients with spatial neglect: pathological effects on subjective straight ahead but not visual open-loop pointing. Neuropsychologia. (2008) 46:1069–80. 10.1016/j.neuropsychologia.2007.11.005 18083203 PMC2600424

[55] Frassinetti F Angeli V Meneghello F Avanzi S Làdavas E. Long-lasting amelioration of visuospatial neglect by prism adaptation. Brain. (2002) 125:608–23. 10.1093/brain/awf056 11872617

[56] Pisella L Rode G Farnè A Boisson D Rossetti Y. Dissociated long lasting improvements of straight-ahead pointing and line bisection tasks in two hemineglect patients. Neuropsychologia. (2002) 40:327–34. 10.1016/S0028-3932(01)00107-5 11684165

[57] Karnath HO Zihl J. S1-Leitlinie Rehabilitation bei Störungen der Raumkognition. (2017). Available online at: https://www.dgn.org/leitlinien/3510-ll-030-126-2017-%09rehabilitation-%09bei-stoerungen-der-raumkognition (accessed May 10, 2021).

[58] Ronga I Sarasso P Raineri F Duhamel JR Becchio C Neppi-Modona M. Leftward oculomotor prismatic training induces a rightward bias in normal subjects. Exp Brain Res. (2017) 235:1759–70. 10.1007/s00221-017-4934-3 28285406

[59] Mancuso M Rosadoni S Capitani D Bickerton WL Humphreys GW De Tanti A. Italian standardization of the Apples Cancellation Test. Neurological Sciences. (2015) 36:1233–40. 10.1007/s10072-015-2088-2 25618236

[60] Basagni B De Tanti A Damora A Abbruzzese L Varalta V Antonucci G. The assessment of hemineglect syndrome with cancellation tasks: a comparison between the Bells test and the Apples test. Neurol Sci. (2017) 38:2171–6. 10.1007/s10072-017-3139-7 28980076

[61] Lucas N Schwartz S Leroy R Pavin S Diserens K Vuilleumier P. Gambling against neglect: unconscious spatial biases inducedby reward reinforcement in healthy people andbrain-damaged patients. Cortex. (2013) 49:2616–27. 10.1016/j.cortex.2013.06.004 23969194

[62] Neppi-Mòdona M Sirovich R Cicerale A Richard N Pradat-Diehl P Sirigu A. Following the gold trail: reward influences on spatial exploration in neglect. Cortex. (2020) 129:329–40. 10.1016/j.cortex.2020.04.027 32559507

[63] Malhotra PA Soto D Li K Russell C. Reward modulates spatial neglect. J Neurol Neurosurg Psychiatr. (2013) 84:366–9. 10.1136/jnnp-2012-303169 23071349 PMC3596771

[64] Schindler I Kerkhoff G Karnath HO Keller I Goldenberg G. Neck muscle vibration induces lasting recovery in spatial neglect. J Neurol Neurosurg Psychiatr. (2002) 73:412–9. 10.1136/jnnp.73.4.412 12235310 PMC1738082

[65] Pisella L Rode G Farnè A Tilikete C Rossetti Y. Prism adaptation in the rehabilitation of patients with visuo-spatial cognitive disorders. Curr Opin Neurol. (2006) 19:534–42. 10.1097/WCO.0b013e328010924b 17102690

